# Inadequate Calcium and Vitamin D Intake Among Japanese Women During the Perinatal Period: A Cross-Sectional Study with Bone Health Assessment

**DOI:** 10.3390/nu17061075

**Published:** 2025-03-19

**Authors:** Ryoko Ichikawa, Megumi Shibata, Yuko Nakura, Katsumi Iizuka, Kazuhiro Uenishi, Takao Sekiya, Atsushi Suzuki, Haruki Nishizawa

**Affiliations:** 1 Department of Obstetrics and Gynecology, School of Medicine, Fujita Health University, Toyoake 470-1192, Aichi, Japan; ryokojjj@fujita-hu.ac.jp (R.I.); kurisen@zg7.so-net.ne.jp (T.S.); nharuki@fujita-hu.ac.jp (H.N.); 2 Department of Endocrinology, Diabetes and Metabolism, School of Medicine, Fujita Health University, Toyoake 470-1192, Aichi, Japan; aslapin@fujita-hu.ac.jp; 3 Nakura Ladies Clinic, Nagoya 465-0017, Aichi, Japan; 4 Department of Clinical Nutrition, School of Medicine, Fujita Health University, Toyoake 470-1192, Aichi, Japan; katsumi.iizuka@fujita-hu.ac.jp; 5 Laboratory of Physiological Nutrition, Kagawa Nutrition University, Saitama 350-0288, Saitama, Japan; uenishi@eiyo.ac.jp

**Keywords:** calcium intake, vitamin D, perinatal period

## Abstract

**Objectives:** We previously reported a high prevalence of hypovitaminosis D (25OHD < 20 ng/mL) in Japanese pregnant women with threatened premature delivery. This study aimed to assess nutritional status and its relationship with bone-related markers and microarchitecture, as measured using quantitative ultrasonography (QUS), in Japanese women during the perinatal period. **Methods:** We recruited Japanese women who had just delivered at Fujita Health University Hospital (n = 103, cesarean/vaginal delivery = 50/53, age 33.9 ± 4.9 years). On the third day postpartum, their calcaneal QUS was measured, and fasting blood samples were collected. **Results:** The mean total energy intake (1720 ± 298 kcal/day) was lower than the normal range for Japanese women (2100 kcal/day). Their calcium intake (446 ± 130 mg/day) was significantly below the recommended daily intake (RDI) in Japan (660 mg/day), with 95% of participants consuming less than the RDI. Although the average vitamin D intake (8.7 ± 1.8 μg/day) met the Japanese RDI (8.5 μg/day), 36% of participants consumed less than the RDI. Calcium intake was positively associated with the intake of lipids, protein, and vitamins A, D, and K. Additionally, calcium intake but not vitamin D intake tended to correlate with serum 25-hydroxyvitamin D (25OHD) levels. The QUS indices showed no significant association with calcium or vitamin D intake. **Conclusions:** During the perinatal period, Japanese women had low calcium intake and relatively low vitamin D intake, accompanied by reduced 25OHD levels. These findings highlight the need for public health recommendations and policies to promote adequate calcium and vitamin D intake during pregnancy.

## 1. Introduction

Osteoporosis is a major cause of immobility and pathological fractures, highlighting the urgent need for measures to extend healthy life expectancy. Bone mineral density (BMD) peaks during adolescence, making it crucial to maintain a proper diet and sunbathing habits from a young age. Insufficient calcium (Ca) and vitamin D intake, along with the excessive consumption of alcohol, caffeine, and salt, have been linked to BMD loss [1,2,3].

Ca is essential for maintaining bone structure. Guidelines recommend a daily Ca intake of 1000–1200 mg for women over 19 years of age [4,5]. In Japan, the recommended daily intake (RDI) for Ca in women is 650 mg [6]. Additionally, the World Health Organization (WHO) advises Ca supplementation after 20 weeks of gestation in populations with low dietary Ca intake to prevent preeclampsia, especially in women at higher risk of hypertension [7,8,9,10]. However, the RDI for Ca during pregnancy remains similar to that for nonpregnant participants. Ca supplementation appears ineffective in reducing hypertensive risks in healthy, nulliparous pregnant women with adequate baseline Ca intake but may lower the risk in those with insufficient intake [9,10].

Vitamin D is derived from food and synthesized in the skin under ultraviolet light. It is converted to 25-hydroxy vitamin D (25OHD) in the liver and then stored. Subsequently, it undergoes 1α-hydroxylation in the kidneys, forming active vitamin D3 (1,25-dihydroxy vitamin D3 or 1,25OHD3). Active vitamin D3 raises blood Ca levels and inhibits parathyroid hormone (PTH) secretion, playing a key role in blood Ca regulation by enhancing Ca absorption in the intestines and reabsorption in the kidneys [11]. Despite its importance in bone metabolism, vitamin D deficiency is prevalent in Japan [12]. Globally, 40–98% of pregnant individuals are estimated to have vitamin D deficiency [13], which is associated with an increased risk of eclampsia, gestational diabetes, gestational hypertension, and fetal growth failure [14,15,16]. We previously reported that hypovitaminosis D (25OHD < 20 ng/mL) is linked to preterm delivery risk in Japanese women [17]. Although evidence of the effects of vitamin D supplementation during pregnancy on fetal development and maternal complications remains limited [18,19], vitamin D deficiency remains a significant global public health concern among pregnant women, in whom inadequate vitamin D levels are associated with adverse maternal and neonatal outcomes [20,21,22].

In this study, we investigated the relationship between nutrition and bone metabolism by measuring serum bone markers, including 25OHD, and calcaneal quantitative ultrasound parameters. Dietary intake frequency surveys of Ca and vitamin D were also conducted in women during the perinatal period.

## 2. Materials and Methods

### 2.1. Participants

Healthy women (n = 103) who delivered at Fujita Health University Hospital were recruited for this study (Table 1). Patients with parathyroid diseases, congenital vitamin D resistance, kidney failure, or other conditions affecting Ca and/or vitamin D homeostasis were excluded. No control group was set for this study. Total energy consumption (excluding alcohol), Ca, vitamin D, and other nutrient intake during pregnancy were estimated using a simple food frequency questionnaire prepared by Uenishi et al. [23]. This FFQ was validated in adult Japanese women aged between 18 and 69 years who were recruited to test reliability and reproducibility [23]. In the 208 women, moderate-to-high Spearman’s correlation coefficients between our FFQ and the conventional diet record method were found in terms of the intake of calcium (r = 0.668), vitamin D (r = 0.413), and energy (r = 0.471). In the 72 women, the coefficients of variance of the four repeated measurements of intakes throughout one year were 14.1% for calcium, 13.6% for vitamin D, and 9.6% for energy. Therefore, we concluded that this FFQ is a useful tool for evaluating the intake of dietary calcium and vitamin D in adult Japanese women. The Review Board for Epidemiology and Clinical Studies of Fujita Health University approved this descriptive study (#HM16-139) (Aichi, Japan), which was conducted in accordance with the 1964 Declaration of Helsinki and its amendments. Written informed consent was obtained from all participants.

### 2.2. Biochemical Measurements

Plasma or serum samples were collected three days after delivery. The seasons of blood sampling were not consistent. Serum 25OHD concentrations were measured using CLIA (DiaSorin, Inc., Stillwater, MN, USA). The inter- and intra-assay coefficients of variation (CV) were 3.7–7.7% and 5.8–10.9%, respectively. Intact parathyroid hormone (iPTH) levels were determined via two-site IRMA (Nichols Diagnostics Institute, San Clemente, CA, USA). Bone-specific alkaline phosphatase (BAP) and tartrate-resistant acid phosphatase 5b (TRACP-5b) were measured using EIA (Osteolinks BAP and TRACP-5b, Sumitomo Bakelite Co., Ltd., Tokyo, Japan). As for intact PTH, its intra-assay CV was 5.2%. Regarding BAP and TRACP-5b, it has been reported that their intra-assay CV values were 2.3% and 5%, and their inter-assay CV values were 3.1% and below 15%, respectively.

### 2.3. Measurement of the Osteo-Sono Assessment Index and Speed of Sound

The calcaneal osteo-sono assessment index (OSI) of the right foot was measured using quantitative ultrasound sonography (QUS) AOS-100SA (FUJIFILM Co., Tokyo, Japan) [24]. The main reason we used QUS instead of dual-X-ray absorptiometry (DXA) is that young Japanese women of childbearing age tend to avoid radiation exposure. Although QUS has lower diagnostic accuracy than DXA, the change in QUS parameters has also been assessed during pregnancy and lactation periods [25]. This device calculates the speed of sound (SOS) and transmission index (TI) as ultrasound waves pass through the calcaneus. The OSI was determined using the following formula: OSI = TI × SOS^2^. The CV values of the QUS parameters of AOS-100SA are below 2.0%.

### 2.4. Statistical Analysis

Data are presented as medians with interquartile ranges or means ± SD. Statistical analyses were performed using StatFlex 7.0.11 software (Artech Co., Ltd., Osaka, Japan). In this study, the significance level (α) was set at 5% (0.05), with a statistical power (1−β) of 80% (0.8) and an expected correlation coefficient (ρ) of 0.3. A two-tailed test was employed, and the required sample size was calculated to be 89. Single-regression analysis was used to examine relationships between nutrient and mineral intake. A *p*-value < 0.05 was considered statistically significant.

## 3. Results

### 3.1. Postdelivery Subjects Have Lower 25OHD Levels

The background characteristics of the enrolled subjects are shown in Table 1. Their mean age was 34 ± 5 years, and their average body mass index after delivery was 23.8 ± 3.0 kg/m^2^. There were 50 cesarean and 53 vaginal deliveries. All subjects had full-term births. Blood tests revealed a marked decrease in 25OHD, reflecting vitamin D storage. Remarkably, all but three had 25OHD levels below 20 ng/dL, although their iPTH levels were not elevated. However, the serum Ca and bone turnover markers (BAP and TRACP-5b) were within normal limits in most subjects.

### 3.2. Ca and Vitamin D Intake During Pregnancy

Using the simplified FFQ method, we examined the total energy and nutrient intake. The average energy intake was slightly lower due to reduced carbohydrate and protein intake, but fat intake was adequate (Supplementary Table S1). In 95% of participants (n = 98), Ca intake was less than 660 mg (RDA). In 34.9% of subjects (n = 36), vitamin D intake was less than 8.5 μg (RDA).

### 3.3. The Relationship Between Food Intake and Ca-Related Micronutrients

**Figure 1:**
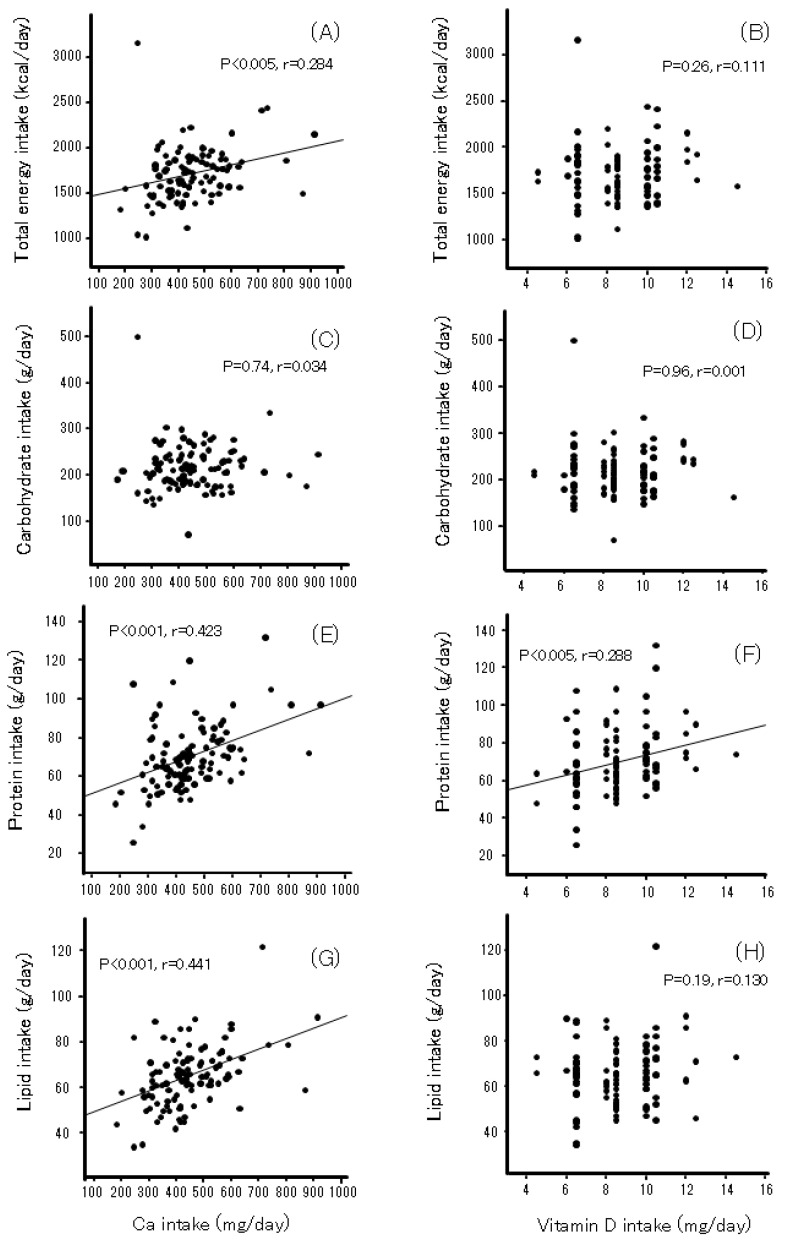
The relationship between calcium (**A**,**C**,**E**,**G**) or vitamin D (**B**,**D**,**F**,**H**) intake and macronutrients in Japanese women during pregnancy and puerperium.

**Figure 2:**
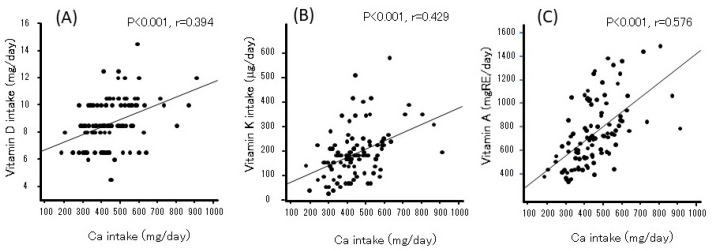
The relationship between calcium and vitamin D (**A**), K (**B**), or A (**C**) intake in Japanese women during the perinatal period.

Ca intake was significantly correlated with total energy, lipids, and protein but not with carbohydrates (Figure 1). Vitamin D intake was associated only with protein. Ca intake was related to lipid-soluble vitamins A, D, and K (Figure 2). Vitamin D intake was correlated with vitamin A (r = 0.285, *p* < 0.005) and vitamin K (r = 0.284, *p* < 0.005).

### 3.4. Ca Intake Was Correlated with 25OHD Levels but Not iPTH Levels

**Figure 3:**
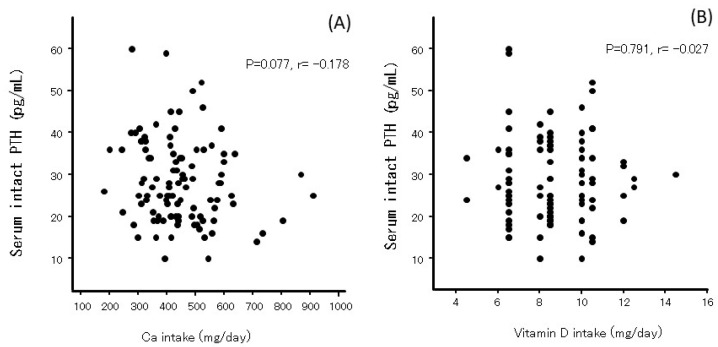
The relationship between calcium (**A**) or vitamin D (**B**) intake with serum iPTH levels just after delivery in Japanese women.

**Figure 4:**
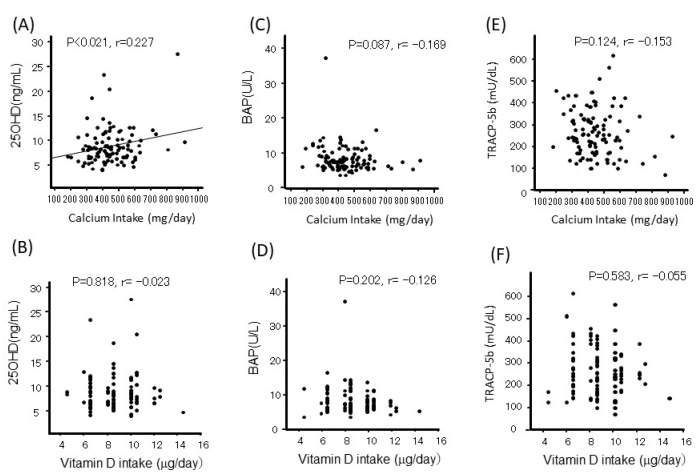
The relationship between calcium (**A**,**C**,**E**) or vitamin D (**B**,**D**,**F**) intake with serum 25OHD (**A**,**B**), BAP (**C**,**D**), or TRACP-5P (**E**,**F**).

Serum iPTH levels were not significantly associated with Ca or vitamin D intake (Figure 3). Serum 25OHD levels were correlated with Ca intake but not with vitamin D intake (Figure 4A,B). Neither Ca nor vitamin D intake affected bone turnover markers such as BAP and TRACP-5b (Figure 4C–F).

### 3.5. Food Intake Was Not Associated with the OSI or SOS of the Calcaneal QUS

**Figure 5:**
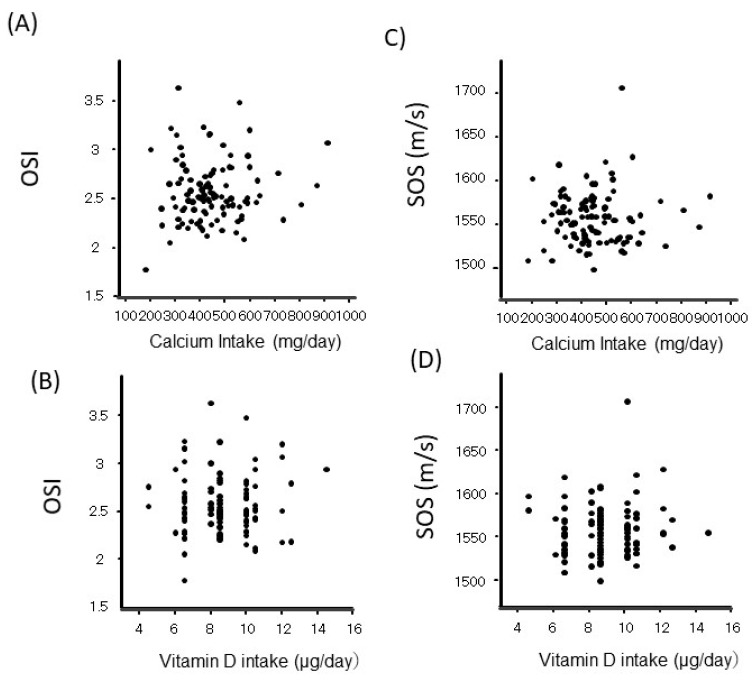
The correlation between calcium or vitamin D intake during the perinatal period with QUS parameters, the osteo-sono assessment index (OSI) (**A**,**B**) or speed of sound (SOS) (**C**,**D**).

Neither the OSI nor SOS of the calcaneal QUS was correlated with Ca or vitamin D intake (Figure 5). The QUS parameters, including SOS and OSI, were not related to serum 25OHD, iPTH, BAP, or TRACP-5b (Table 2).

## 4. Discussion

In the present study, we found that many pregnant Japanese women had hypovitaminosis D and Ca intake below the RDI. Most participants had 25OHD levels below the standard. Ca intake was correlated with serum 25OHD but not with Ca levels. Biomarkers related to bone metabolism and QUS parameters were not correlated with Ca or vitamin D intakes. Thus, hypovitaminosis D and low Ca intake did not have a direct negative impact on bone metabolism in the short term.

The lack of correlation between Ca or vitamin D intake and bone markers is to be expected. Blood Ca levels are tightly regulated by PTH, PTH-related protein (PTHrP), and active vitamin D, meaning they are independent of Ca intake [26]. Vitamin D deficiency reduces Ca absorption, increasing PTH secretion. During pregnancy, placental PTHrP suppresses PTH secretion, which resumes postpartum. Lactation increases PTHrP, further suppressing PTH. Blood tests were conducted on the third day postpartum, making it difficult to assess optimal serum iPTH levels. Hence, vitamin D sufficiency cannot be accurately evaluated using iPTH levels alone.

Previously, we showed that Ca intake correlates with lipid and protein but not carbohydrate intake in Japanese patients with type 2 diabetes [27]. In this study, Ca intake was also weakly but significantly correlated with total energy, protein, and lipid intake in pregnant women without diabetes. Vitamin D intake was associated only with protein intake. Ca-rich foods include fish, dairy products, soy, and vegetables, while vitamin D is found in mushrooms, seafood, and eggs [28]. Protein-rich foods may contribute to both Ca and vitamin D intake.

We observed that most Japanese women at delivery had hypovitaminosis D, consistent with reports of vitamin D deficiency in young Japanese women [12,29]. Sun exposure likely affects serum 25OHD levels, and variations in outdoor activity during pregnancy may contribute to this deficiency [30]. Although the seasons in which the blood samples were taken in this study were not consistent, the subjects consistently showed low vitamin D levels. PTHrP enhances the conversion of 25OHD to 1,25OHD3, possibly lowering 25OHD levels [26,31]. Increased plasma volume during pregnancy could further reduce 25OHD. Despite low vitamin D intake, 25OHD levels did not correlate with vitamin D intake. Maternal vitamin D supplementation has been shown to improve 25OHD levels and bone mass in newborns born in winter [32], reduce urinary CTX (a bone resorption marker), and enhance postpartum bone density [33]. Although the evidence regarding the role of vitamin D supplementation in reducing pregnancy complications remains inconclusive, supplementation has been shown to improve maternal 25-hydroxyvitamin D levels and modestly increase neonatal birth weight and length [22]. Furthermore, maternal vitamin D supplementation has been reported to increase BMD in children at 4 years old compared with a placebo [34].

Vitamin D deficiency remains a significant global public health concern, affecting pregnant women [12,20,21,22]. The current guidelines for obstetrical practice in Japan do not include vitamin D intake requirements for pregnant women [35]. As fish is the primary dietary source of vitamin D in Japan, promoting fish consumption might be effective. On the other hand, dietary guidance limits fish consumption to avoid mercury, which may inadvertently reduce vitamin D intake in pregnant women [36]. To address low Ca and vitamin D intake, foods rich in both, such as small fish and shrimp, should be recommended. Standardized supplementation protocols, systematic food fortification programs, and targeted educational interventions could reduce the risks associated with vitamin D deficiency and optimize maternal and neonatal health outcomes.

Several limitations of this study should be noted. First, this is a cross-sectional observational study, and longitudinal studies are needed to evaluate the impact of dietary intake on bone health. Second, nutrient intake was assessed using a seven-day FFQ, which may not accurately reflect daily vitamin D intake due to its variability [37]. In addition, the validation of all trace minerals was not completed for this FFQ. This might explain the lack of a significant correlation between vitamin D intake and serum 25OHD levels. Third, although DXA is a more direct method of assessing bones than QUS, it involves the risk of X-ray exposure. Fourth, the effect of PTHrP, which was not measured, should be considered. Fifth, the duration of sunlight exposure and seasonal variations were not assessed in this population. Sixth, the study involved 103 participants from a single center in Aichi Prefecture; thus, larger-scale studies targeting more pregnant women across multiple regions are needed.

## 5. Conclusions

We examined Ca and vitamin D intake in pregnant subjects postdelivery. Many showed low Ca and vitamin D intake, with low 25OHD levels. These findings highlight the need for public health recommendations and policies to promote adequate calcium and vitamin D intake during pregnancy. Strengthening nutritional education, fortifying dietary guidelines, and implementing population-wide interventions could contribute to optimizing maternal and neonatal bone health.

## Figures and Tables

**Table 1:** Clinical manifestation of this study.

	Total (n = 103)	Normal Range	Number of Cases	
			Below Lower Limit	Above Upper Limit
Age (years old)	34 ± 5			
Cesarean/vaginal delivery	50/53			
Gestation period (days)	281 ± 6	259–294		
Body mass index (kg/m^2^)	23.8 ± 3.0	18.5–25	2 (1.9%)	29 (28.2%)
Newborn weight at birth (g) *	2968 ± 419	2500–4000	11(10.7%)	1 (1.0%)
Serum calcium (corrected) (mg/dL)	9.5 ± 0.4	8.2–10.0	0	9
Serum phosphate (mg/dL)	4.1 ± 0.5	2.4–4.1	0	50 (48.5%)
Serum 25OHD (ng/mL)	8.9 ± 3.7	20	99 (96.1%)	0
Serum blood urea nitrogen (mg/dL)	9.3 ± 2.5	5.0–12.0 ^#^	1 (1.0%)	10 (9.7%)
Serum creatinine (mg/dL)	0.5 ± 0.1	0.39–0.73	4 (3.9%)	0
Serum albumin (g/dL)	2.8 ± 0.4	2.5–4.0 ^#^	0	0
Serum intact PTH (pg/mL)	28 ± 10	15−65	3 (2.9%)	0
Serum BAP (U/L)	8.3 ± 3.9	2.9–14.5	0	2 (1.9%)
Serum TRACP-5b (mU/dL)	264 ± 106	120–420	3 (2.9%)	4 (3.9%)

25OHD: 25-hydroxy vitamin D; PTH: parathyroid hormone; BAP: bone-specific alkaline phosphatase; TRACP-5b: tartrate-resistant acid phosphatase-5b. ^#^ Normal range during pregnancy; * 102 cases, excluding cases of twin pregnancies.

**Table 2:** Serum parameters and bone indices assessed using calcaneal quantitative ultrasonography.

	SOS		OSI	
	r	*p*-Value	r	*p*-Value
Intact PTH	0.028	0.773	0.094	0.345
25OHD	−0.150	0.130	−0.051	0.612
BAP	0.133	0.180	0.132	0.185
TRACP-5b	−0.054	0.591	−0.020	0.841

PTH: parathyroid hormone; 25OHD: 25-hydroxy vitamin D; BAP: bone-specific alkaline phosphatase; TRACP-5b: tartrate-resistant acid phosphatase-5b; SOS: speed of sound; OSI: osteo-sono assessment index.

## Data Availability

The original contributions presented in the study are included in the article and further inquiries can be directed to the corresponding authors.

## Supplementary Materials

The following supporting information can be downloaded at: https://www.mdpi.com/article/10.3390/nu17061075/s1, Table S1: Amount of food intakes in study subjects.

## References

[1] Sahni S., Mangano K.M., Tucker K.L., Kiel D.P., Casey V.A., Hannan M.T. (2014). Protective association of milk intake on the risk of hip fracture: Results from the Framingham Original Cohort. J. Bone Miner. Res..

[2] American Geriatrics Society Workgroup on Vitamin D Supplementation for Older Adults (2014). Recommendations abstracted from the American Geriatrics Society consensus statement on vitamin D for prevention of falls and their consequences. J. Am. Geriatr. Soc..

[3] Cosman F., de Beur S.J., LeBoff M.S., Lewiecki E.M., Tanner B., Randall S., Lindsay R. (2014). National Osteoporosis Foundation. Clinician’s guide to prevention and treatment of osteoporosis. Osteoporos. Int..

[4] Tang B.M., Eslick G.D., Nowson C., Smith C., Bensoussan A. (2007). Use of calcium or calcium in combination with vitamin D supplementation to prevent fractures and bone loss in people aged 50 years and older: A meta-analysis. Lancet.

[5] Yao P., Bennett D., Mafham M., Lin X., Chen Z., Armitage J., Clarke R. (2019). Vitamin D and calcium for the prevention of fracture: A systematic review and meta-analysis. JAMA Netw. Open.

[6] Dietary Reference Intake for Japanese. https://www.mhlw.go.jp/content/001151422.pdf.

[7] WHO Antenatal Care Recommendations for a Positive Pregnancy Experience Nutritional Interventions Update: Multiple Micronutrient Supplements During Pregnancy. https://www.who.int/publications/i/item/9789240007789.

[8] Dietary Guidelines for Americans 2020–2025. https://www.dietaryguidelines.gov/sites/default/files/2021-03/Dietary_Guidelines_for_Americans-2020-2025.pdf.

[9] Hofmeyr G.J., Lawrie T.A., Atallah Á.N., Torloni M.R. (2018). Calcium supplementation during pregnancy for preventing hypertensive disorders and related problems. Cochrane Database Syst. Rev..

[10] Buppasiri P., Lumbiganon P., Thinkhamrop J., Ngamjarus C., Laopaiboon M., Medley N. (2015). Calcium supplementation (other than for preventing or treating hypertension) for improving pregnancy and infant outcomes. Cochrane Database Syst. Rev..

[11] Vautour L., Goltzman D., Bilezikian J.P. (2019). Regulation of calcium Homeostasis. Primer on the Metabolic Bone Diseases and Disorder of Mineral Metabolism.

[12] Asakura K., Etoh N., Imamura H., Michikawa T., Nakamura T., Takeda Y., Mori S., Nishiwaki Y. (2020). Vitamin D status in Japanese adults: Relationship of serum 25-hydroxyvitamin D with simultaneously, measured dietary vitamin D intake and ultraviolet ray exposure. Nutrients.

[13] van Schoor N.M., Lips P. (2011). Worldwide vitamin D status. Best Pract. Res. Clin. Endocrinol. Metab..

[14] Aghajafari F., Nagulesapillai T., Ronksley P.E., Tough S.C., O’Beirne M., Rabi D.M. (2013). Association between maternal serum 25-hydroxyvitamin D level and pregnancy and neonatal outcomes: Systematic review and meta-analysis of observational studies. BMJ.

[15] Cannell J.J. (2017). Vitamin D and autism, what’s new?. Rev. Endocr. Metab. Disord..

[16] Dawodu A., Akinbi H. (2013). Vitamin D nutrition in pregnancy: Current opinion. Int. J. Womens Health..

[17] Shibata M., Suzuki A., Sekiya T., Sekiguchi S., Asano S., Udagawa Y., Itoh M. (2011). High prevalence of hypovitaminosis D in pregnant Japanese women with threatened premature delivery. J. Bone Miner. Metab..

[18] Mohammad-Alizadeh-Charandabi S., Mirghafourvand M., Mansouri A., Najafi M., Khodabande F. (2015). The effect of vitamin D and calcium plus vitamin D during pregnancy on pregnancy and birth outcomes: A randomized controlled trial. J. Caring Sci..

[19] Karamali M., Asemi Z., Ahmadi-Dastjerdi M., Esmaillzadeh A. (2016). Calcium plus vitamin D supplementation affects pregnancy outcomes in gestational diabetes: Randomized, double-blind, placebo-controlled trial. Public Health Nutr..

[20] Fiscaletti M., Stewart P., Munns C.F. (2017). The importance of vitamin D in maternal and child health: A global perspective. Public Health Rev..

[21] Hemmingway A., O’Callaghan K.M., Hennessy Á., Hull G.L.J., Cashman K.D., Kiely M.E. (2018). Interactions between Vitamin D Status, Calcium Intake and Parathyroid Hormone Concentrations in Healthy White-Skinned Pregnant Women at Northern Latitude. Nutrients.

[22] Pérez-López F.R., Pasupuleti V., Mezones-Holguin E., Benites-Zapata V.A., Thota P., Deshpande A., Hernandez A.V. (2015). Effect of vitamin D supplementation during pregnancy on maternal and neonatal outcomes: A systematic review and meta-analysis of randomized controlled trials. Fertil Steril..

[23] Uenishi K., Ishida H., Nakamura K. (2008). Development of a simple food frequency questionnaire to estimate intakes of calcium and other nutrients for the prevention and management of osteoporosis. J. Nutr. Sci. Vitaminol..

[24] Tsuda-Futami E., Hans D., Njeh C.F., Fuerst T., Fan B., Li J., He Y.Q., Genant H.K. (1999). An evaluation of a new gel-coupled ultrasound device for the quantitative assessment of bone. Br. J. Radiol..

[25] Hellmeyer L., Hahn B., Fischer C., Hars O., Boekhoff J., Maier J., Hadji P. (2015). Quantitative ultrasonometry during pregnancy and lactation: A longitudinal study. Osteoporos. Int..

[26] Kovacs C.S., Kronenberg H.M., Bilezikian J.P. (2019). Pregnancy and lactation. Primer on the Metabolic Bone Diseases and Disorder of Mineral Metabolism.

[27] Tomastu E., Ninomiya E., Ando M., Hiratsuka I., Yoshino Y., Sekiguchi-Ueda S., Shibata M., Ito A., Uenishi K., Suzuki A. (2016). Nutritional status of calcium and other bone-related nutrients in Japanese type 2 diabetes patients. Osteoporos. Sarcopenia.

[28] Standard Table of Food Composition in Japan -2015- (Seventh Rebased Version). https://www.mext.go.jp/en/policy/science_technology/policy/title01/detail01/1374030.htm.

[29] Iizuka K., Sato H., Kobae K., Yanagi K., Yamada Y., Ushiroda C., Hirano K., Ichimaru S., Seino Y., Ito A. (2023). Young Japanese Underweight Women with “Cinderella Weight” Are Prone to Malnutrition, including Vitamin Deficiencies. Nutrients.

[30] Fazzi C., Saunders D.H., Linton K., Norman J.E., Reynolds R.M. (2017). Sedentary behaviours during pregnancy: A systematic review. Int. J. Behav. Nutr. Phys. Act..

[31] Ardawi M.S., Nasrat H.A., BA’Aqueel H.S. (1997). Calcium-regulating hormones and parathyroid hormone-related peptide in normal human pregnancy and postpartum: A longitudinal study. Eur. J. Endocrinol..

[32] Cooper C., Harvey N.C., Bishop N.J., Kennedy S., Papageorghiou A.T., Schoenmakers I., Fraser R., Gandhi S.V., Carr A., D’Angelo S. (2016). Maternal gestational vitamin D supplementation and offspring bone health (MAVIDOS): A multicentre, double-blind, randomised placebo-controlled trial. Lancet Diabetes Endocrinol..

[33] Curtis E.M., Parsons C., Maslin K., D’Angelo S., Moon R.J., Crozier S.R., Gossiel F., Bishop N.J., Kennedy S.H., Papageorghiou A.T. (2021). Bone turnover in pregnancy, measured by urinary CTX, is influenced by vitamin D supplementation and is associated with maternal bone health: Findings from the Maternal Vitamin D Osteoporosis Study (MAVIDOS) trial. Am. J. Clin. Nutr..

[34] Curtis E.M., Moon R.J., D’Angelo S., Crozier S.R., Bishop N.J., Gopal-Kothandapani J.S., Kennedy S.H., Papageorghiou A.T., Fraser R., Gandhi S.V. (2022). Pregnancy Vitamin D Supplementation and Childhood Bone Mass at Age 4 Years: Findings from the Maternal Vitamin D Osteoporosis Study (MAVIDOS) Randomized Controlled Trial. J. Bone Miner. Res. Plus.

[35] Guidelines for Obstetrical Practice in Japan: Japan Society of Obstetrics and Gynecology and Japan Association of Obstetricians and Gynecologists 2023 Edition. https://www.jsog.or.jp/activity/pdf/gl_sanka_2023.pdf.

[36] Taylor C.M., Emmett P.M., Emond A.M., Golding J. (2018). A review of guidance on fish consumption in pregnancy: Is it fit for purpose?. Public Health Nutr..

[37] Iizuka K., Deguchi K., Ushiroda C., Yanagi K., Seino Y., Suzuki A., Yabe D., Sasaki H., Sasaki S., Saitoh E. (2024). A Study on the Compatibility of a Food-Recording Application with Questionnaire-Based Methods in Healthy Japanese Individuals. Nutrients.

