# Supplementary material for: Inadequate Calcium and Vitamin D Intake Among Japanese Women During the Perinatal Period: A Cross-Sectional Study with Bone Health Assessment
Source: Nutrients. 2025 Mar 19;17(6):1075. doi: 10.3390/nu17061075 (PMC11944662; doi:10.3390/nu17061075)
Supplement: Supplementary file 1 [file nutrients-17-01075-s001.zip › nutrients-3525542-supplementary.pdf]

Supplementary Table S1 Amount of food intakes in study subjects

		DG	Average ± SD	Number of cases showing values below the lower limit
Energy (kcal/day)		2100	1720 ± 298	95 (92.2%)
Macronutrients	Protein (g/day)	78-105	70 ± 17	77 (74.8%)
	Lipid (g/day)	47-70	65 ± 14	10 (9.7%)
	Carbohydrate (g/day)	262-341	217 ±50	86 (83.5%)
Micronutrients	Ca (mg/day)	660#	446 ± 130	98 (95.1%)
	Vitamin A (μgRE/day)	1100#	742 ± 279	92 (89.3%)
	Vitamin D (μg/day)	8.5*	8.7 ± 1.8	36 (35.0%)
	Vitamin K (μg/day)	150*	192 ± 101	33 (32.4%)

DG: Tentative dietary goal for preventing life-style related disease
* AI: Adequate intake
# RDA: Recommended dietary allowance
